# On the Curative Influence of the Climate of Pau, and the Mineral Waters of the Pyrenees, on Disease, &c.

**Published:** 1844-01-01

**Authors:** 


### On the Curative Influence of the Climate of Pau, and the Mineral Waters of the Pyrenees, on Disease; with Descriptive Notices, &c. &c. By *A. Taylor*, M.D. 8vo. pp. 342. Parker, London, 1842.

The Waters of the Pyrenees are little, if at all, inferior to some of the most famous Spas of Germany—the scenery, nearly equal to that of the Alps—the distance from London not more than the journey to Carlsbad—the language much better, and more generally understood than the German—and yet 500 English go to the German Springs for one that goes to Bagnères de Bigorre, Barréges, or the Cauterets! There are various reasons for this disparity. The waters of the Pyrenees have not been "written up" like those of Nassau, Bavaria, and Bohemia. They are, indeed, so little known to the profession in England, that very few can give their patients any opinion as to their composition or qualities—much less their applicability to individual complaints. Another reason of the preference alluded to, is the romantic portal or avenue to the German Spas—the lovely Rhine—forming a remarkable contrast with that "long rough road," which wearies the eye and half dislocates the joints of the Valetudinary Pilgrim in pursuit of Health, in whatever direction he may travel in France. But there is yet another reason—more perhaps of a moral than a physical character. The French may be, and doubtless are, a most polite people; but they hate the English, and every thing about the English—except their money! The English know this—and feel this, but still they will spend their money, their time, and their morals in Paris—ostensibly for economy, but really to command more luxuries for the same sum than they could do in their native, but distressed land! Nevertheless, when out of health, the Briton naturally prefers those springs which rise in countries congenial to his own, and whose inhabitants derive their origin from the same stock or race as himself. Difficult as the German language appears to him, he finds, at every step, the most remarkable traces of affinity between it and his native tongue. The invalid, therefore, finds himself, as it were on a friendly soil, and among relations, rather than neighbours of doubtful—or rather of hostile feelings towards him. But there may be times and circumstances, and even necessities, for visiting certain of the Gallic Spas, especially those among the Pyrenees, and a work like the present, was wanted to make the English medical man as well acquainted generally with the Spas of France, as he is now with those of Trans-Rhenish celebrity.

Dr. Taylor's work is divided into not less than nineteen Chapters, of very various interest to the English reader. Some of them we shall pass over very cursorily, whilst to others we shall dedicate more attention.

### Chap. I.—Introductory.

We are informed that, for twenty years past, Pau has been gradually, but steadily, acquiring celebrity "among an unbroken succession of visitors," who have derived benefit from the salubrity of its climate, but still it wanted an impartial historian. Some years residence there—occasional visits to the mineral waters in the vicinity—and the assistance of professional friends, have enabled Dr. Taylor to undertake this task with some degree of confidence. PAU was celebrated in ancient times for the salubrity of its air, and—

"At the present day, rigid statistics stamp it as *the* province of France, possessing, *par excellence*, a climate calculated to promote health, and to subdue certain kinds of disease, both organic and functional." 7.

Our author avers that, in addition to the air, "the waters of the neighbouring mountains are of surpassing efficacy in the treatment of membranous disease, and a variety of symptoms that follow in its train."

### CHAP. II.—HINTS TO INVALIDS, &c.

The easiest route is by Southampton, Havre, and Bourdeaux, by steam. The voyage is generally useful in most complaints for which the climate of PAU is recommended. The distance from Bourdeaux to PAU is 125 miles, and is performed in two or three days. The journey should not be undertaken later than the first of September, by those who wish to pass the Winter at PAU, who should repair to that place well supplied with flannel and warm clothing; for there is a vast difference among the Pyrenees between the sun and the shade. Residences with southern aspects, are greatly preferable to all others there. Visitors from Italy approach by Toulouse—from England by Bourdeaux. The towering Pyrenees are seen by both parties at a long distance off. On approaching PAU, and on reaching the top of a hill, a magnificent view bursts on the eye of the traveller. Immediately beneath, is a valley, with a little world of villages, hamlets, vineyards, and streams within itself—rich in fertility, and lighted up by a brilliant sun. Behind the valley rises the vast line of Pyrenees, in Nature's grandest forms. PAU is the capital of the ancient Province of Bearne, and now the chief city of the Basses Pyrenees, distant about 20 miles from the nearest part of the Pyrenean Chain, and commanding a fine view of them as we look up the valley of the Gave, towards the Pic de Bagnères. The town contains about 14,000 inhabitants, and is well built, the natives being polite and good-natured. Carriages are to be had in abundance, and trottoirs are extending along the streets. The town is built on a terrace overhanging the river 150 feet, and facing the Pyrenees. It is sheltered on the north by the *Landes* of the Pont-long, which gradually ascend to the distance of 15 miles from PAU. The north-winds, therefore, pass over the town at some height, attracted by the mountains to the south. "The clouds may be often seen quietly sailing onwards, when the leaves are unmoved on the lower level." There is a curvelinear head-land to the west that proves a shelter in that direction. PAU is embraced on the south and south-west by the amphitheatre of Turançon, which screens it and its environs from the only bad weather-quarter—the west and north-west. The east and south-east winds are hardly even felt at PAU, except as bringing dry and warm weather. Thus, at PAU, one enjoys a stillness of atmosphere so complete as to leave a doubt respecting the quarter from which the wind really blows.

The season here may be said to commence on the first of September, and to continue till the first of June. There are here at least one hundred suites of apartments, tolerably well furnished, independently of small lodging-houses and the hotels. Some of these suites offer accommodations for 12 or 16 persons, and are moderate in price. The inhabitants understand the tastes and habits of the English, and take care to please them if possible. The English Service is performed twice a day on Sundays.

Although more rain falls in Pau than in London, yet the number of rainy days in the former is much fewer. The rains at this place fall in large and sudden quantities—often after sunset, and are soon absorbed by the soil. There are few days, therefore, in which people in health, or even invalids, may not take exercise in the middle of the day. The mean temperature (9—1, and five o'clock), of Autumn, is 62°—Winter, 45°—Spring, 58°.

The west-wind brings on its soft and soothing wings volumes of Atlantic vapour, to be expended in the absorption of the excess of electricity accumulated during the south and south-east wind that blew previously. There are several circumstances, according to the testimony of those who have resided at Pau, which render it a desirable *sejour* for invalids. The atmosphere, when it does not rain, is dry, and the weather fine, without either fogs or piercing winds. The Spring is characteristically mild and exempt from cold winds. It is thus favourable to those who are affected with tracheal or bronchial complaints. The following passage from the Author of a Summer and Winter in the Pyrenees, is characteristic.

"At the foot of a woody range of high ground, forming the promenade above described, (the Parc,) runs the broad shallow river Gave, with a perpetual low murmur that lulls the senses to repose. It is, in fact, the only sound we hear; for there is so little wind in this climate that not a leaf is seen to move, and we, therefore, distinguish at a greater distance the toll of the matin and vesper bell in the neighbouring villages, and the tinkling sounds which tell when the flocks are led to and from the fields. There appears at first to be a sort of mystery in this universal stillness. It seems like a pause in the breath of nature, a suspension of the general throb of life; and we almost feel as if it must be followed by that shout of joy which the language of poetry has so often described, as the grateful response of nature for the blessings of light and life; and never surely could this response be offered more appropriately than from such a scene as this rich and fertile land presents." 50.

The mean annual temperature of Pau is 6° higher than London—$4\frac{1}{2}$° higher than Penzance—3° lower than Marseilles, Nice, and Rome—17° lower than Madeira.

On market days at Pau, a great number of the neighbouring peasantry flock into the town, and their appearance bespeaks health and comfort. Dr. Taylor avers, that the pulse is slower among the Bearnois than among other people, which he attributes to the placid and unirritating state of the atmosphere. In respect to the diseases affecting the native population, it is said that there are no predominant ones—that Pau is exempt from endemic or epidemic maladies—that scrofulous and tuberculous diseases are comparatively rare—that rheumatism is as common as in most other places—that bronchitis is not unfrequent during the Winter and Spring, but not of so acute a character as in England—that glandular and mesenteric diseases are rare, but cerebral congestion and gastro-intestinal irritation are the grave maladies among the native children—that inflammatory diseases are of a mild type. The mortality in PAU is about one in forty-five annually—five less than in London, and twenty-two less than in Vienna.

In respect to the influence of this climate on the health of English sojourners, Dr. Taylor observes, that it is sedative to those who are well, diminishing nervous energy and arterial circulation, but increasing the tendency to venous congestion. The healthy stranger, therefore, complains of languor and listlessness, with sense of fulness about the head and chest, and oppression at the pit of the stomach. Under these circumstances, exposure to the sun's rays is dangerous, and a mild alterative aperient is necessary, with or without the shower-bath.

The mortality among the English population, for four years preceding 1842, was, according to Dr. Taylor, very low, considering the proportion of invalids—being only one in 65 to 70. The ratio of deaths from chest complaints was one in 150. The climate of PAU, like that of Nice or Italy, or Madeira, is more valuable as a preventive than as a cure for pulmonary affections. According to Dr. Taylor, the following are the indications.

"1. As the liability to strumous and lymphatic diseases in children is more a predisposition than an hereditary *germ*, it may not be illogical to infer that a climate, in which the native population is very sparingly visited with these affections, is one, if by times resorted to, calculated to repress the bias to their development.

"2. Wherever among children or adults, whether of suspected strumous taint or not, there is the tumid abdomen, the uncertain appetite, the wasted condition, the waning strength, and the hurried small sharp pulse, indicating the commencement of mesenteric disease.

"3. Infantile predisposition to cerebral inflammation, false croup, spasmodic asthma, and to inflammatory attacks in general.

"4. The climate acts beneficially also in discouraging the generation of tuberculous matter, by diminishing irritation of the mesenteric glands and lacteal system, and consequently preventing its deposition in different structures.

"5. In checking tuberculous deposits from coming to maturity, by diminishing the quantity of the pulse and the frequency of respiration; and thus, in the lungs for instance, preventing inflammatory fluxion to the tuberculous points, which, as foreign bodies, under circumstances which increase the circulation, are liable to take on a softening process.

"6. In all tendencies to disease depending on a nervo-sanguineous temperament, such as nervous headache, convulsive disorders in the same temperament, and liabilities to periodic inflammatory action: all aberrations of secretion depending on too high a state of irritability of the secreting organ.

"7. Indeed the predispositions which are favourably influenced by the climate of Pau, may be summed up in one general principle:—viz., wherever it depends upon increased nervous and arterial action, permanently produced either by temperament or by some cause leading to more active disease.

"The morbid condition of the system, favourably influenced by the climate, is that depending on continued inflammatory irritation of some important organ, and which, either independently, or by sympathy, is being prepared for a disorganised state incompatible with life. As the mode of action of the climate is that of a direct sedative, modifying, and altogether reducing, where this is possible, irritation, both nervous and vascular, it is evident that the chief benefit derivable from it, is to be looked for, *par excellence,* in that stage of disease preceding, and, if unchecked, leading to dangerous organic mischief." 87.

This is more particularly the case in gastro-intestinal affections. The climate of PAU is supposed to act beneficially in cases where tubercles exist in a passive state in the lungs. In those inflammations of the bronchi and trachea, to which public speakers are liable now-a-days, the climate in question is said to exercise its highest powers. Dr. Taylor avers, contrary to the opinion of Sir James Clarke, that rheumatism is not aggravated by the climate of PAU, especially among strangers, though it is common among the inhabitants. Dr. T. acknowledges, however, that there are disorders to which the climate of PAU is not favourable. These are, atonic dyspepsia, and its long train of attendant phenomena—broken-down constitutions, from residence in hot climates—catarrhus senilis, and chronic bronchitis, with loss of tone and excess of expectoration—chronic rheumatism, complicated with atonic gout, and attended with debility of the digestive organs—apoplectic tendencies, depending upon congestion of the brain or other organs in leucophlegmatic habits—chlorosis, from atony and passive congestion of the uterine system—" all diseases where there are congestion of the venous system, and diminished nervous energy." The qualities of the climate of PAU are thus summed up by Dr. Taylor.

" 1st. Its soil being gravelly to a great depth, absorbs most readily any quantity of moisture that may fall, so that there is no stagnant water to be re-absorbed into the atmosphere. 2nd. From the topographic features of the country surrounding Pau, it is almost completely shielded from wind, so much so that for weeks it is difficult to indicate the point from which the wind blows. 3rd. From the bias which the wind has to blow from the south-west, west, and north-west, we find, that, if in the morning, it proceeds from the east, at mid-day from the south, that more or less electric matter is thus generated. But the decline of the sun, in this case, seems to solicit the wind from the west, and to invite Atlantic vapour to absorb the excess of electric fluid, which, when not so expended, as in Nice, and in the south-east of France, exerts so irritating an influence on nervo-sanguineous temperaments, and on inflammatory affections of membranes and glands. 4th. Although we have considerable atmospheric variations at Pau, still, from the great absence of agitation of the air, these variations pass innocuously over the invalid. Indeed, the human system, in health and disease, seems to partake of the same tranquillity which reigns abroad in the external world. 5th. The marked absence of free communicable moisture also in the atmosphere, as indicated by the hygrometer, is a state highly favourable to the alleviation and cure of diseases, the produce of exciting and humid climates. 6th. Acting on persons in health, the climate brings down the standard of tone, and modifies the natural temperament, the sanguine making a move towards the phlegmatic, and the choleric towards the melancholic. On the same principle, no doubt it is, that diseases of a mixed, nervous, and inflammatory character, come to have their symptoms modified and frequently subdued. That kind of functional derangement of a tonic irritable type, which paves the way to severe organic lesion, it will be seen, from what has been previously said, is the state of things for the alleviation and cure of which the qualities of the climate are most suited, as well as in preventing the development of pending predispositions. The Author also has occasionally observed continual irritation of the trachea and bronchi, as well as of pulmonary tubercles, accompanied with purulent expectoration, quick pulse, hectic fever, and emaciation, to be subdued by a transfer of action to the liver; a manageable state of congestion of this organ being the expense for the relief of symptoms threatening to life. Three or four instances of this mode of amelioration have come under his notice during the last few years, where the patients are now entirely recovered from the pulmonary symptoms. In mesenteric irritation of children, also, there is often the same kind of beneficial transfer, most chiefly to the liver, and occasionally to the hæmorrhoidal vessels." 97.

Dr. Taylor winds up this part of the work by a kind of parallel between the climate of PAU and some of the principal resorts of invalids in other parts of the world. Thus, in the south-east of France (Provence and Languedoc), less rain falls than in any other part of Europe, and a complete drought for months in succession is not unusual. In the Winter it is colder and drier than in the south-west. The Bize and Mistral Winds, too, are very annoying and injurious to invalids—more especially, to pulmonary ones. Moving on towards NICE, we perceive features entirely opposite to those of PAU. There the easterly wind sets in with the "blood-red moon" of March, and is severely felt by almost all people in delicate health. Dr. Farr has feelingly warned pulmonary invalids to leave Nice in the Spring—the great objection to its climate being its dryness, and the exciting and irritating nature of its atmosphere rendering it injurious to all complaints requiring a sedative to the nervous and vascular systems. The natives of Nice are more subject to diseases of an acute inflammatory type than those of PAU. According to Sir James Clark there are very few cases of pulmonary consumption, or even a tendency to it, which will be benefited by the climate of Nice. It is found that the mortality at Nice is one in 31, whereas at PAU it is one in 45, as we stated before.

But the climate under consideration approximates as much to that of Rome, as it differs from that of Nice. It is fully as sedative as the air of the Eternal City.

### MINERAL WATERS OF THE PYRENEES.

This lofty and romantic Alpine chain of mountains, that separate France from Spain, pour out, especially on the northern side, many mineral springs—sulphureous—saline—and chalybeate. We shall notice the chief of these.

### I. BAGNERES DE BIGORRE.

This is situated at the base of the Pyrenees, 35 miles from PAU, in a south-eastern direction, and at the entrance of a smiling valley, being neither in the mountains, nor yet in the open country. The temperature is not so high as at PAU, nor subject to such reductions as we experience at an early period of the season in the other watering-places more distant from the plain. The mean temperature of the fine season (June to October) is about 64° of Fahrenheit. Thus a patient may leave Pau for Bagnères in the month of May, while it might be dangerous for him to take up his abode among the mountains at this early period, or even for five or six weeks afterwards. The waters here, being less potent than those that are higher up the Pyrenees, are good preparatives for the latter in many disorders and diseases. The antiquity of these waters is traced up to the Siege of Troy, or even the Wars of the Giants and Gods. The Romans have left records of their acquaintance with these springs—and that is antiquity enough.

"There is perhaps no town in France which has greater attractions at first sight than Bagnères. As you approach it from Tarbes, which is distant thirteen miles, you have at first a very fruitful plain, then richly-wooded coteaux advancing in gradual undulation, encircling Bagnères, then mountain piled above mountain; and towering over all, in the elevated horizon, the Pic de Midi, at a height of 10,000 feet, stretching over the less elevated mountains in its foreground, as if peeping into the town." 223.

On entering the town itself, we are agreeably surprised to see well-kept Macadamized streets, with neat whitewashed houses on both sides. The houses are commodious—the gardens numerous—its promenades command magnificent prospects of mountain and precipice—wood and water. The town contains a fixed population of 8,000 souls, exclusive of visitors; 4,000 of which it can accommodate in the season (June to October). It is 1,600 feet above the level of the sea, and snugly nestling at the very feet of the Pyrenees. The following observations of Mr. Inglis may be introduced here:—

"It has been said of Bagnères, that it is a town where pleasure has raised her altars besides those of Esculapius; and this is true, for it is only at Bagnères, among all the watering-places of the Pyrénées, that that kind of pleasure is to be found, which is usually sought for at a watering-place. Bagnères is, for this reason, by far the most frequented of the baths, because it is not resorted to by invalids only, but also by two other kinds of visitors, those whose slight ailments are compatible with the pursuits of pleasure, and those who are driven by the heats of Summer, from the plains of France to the mountain air of the Pyrénées. Among this latter class may be ranked the great majority of the English who reside at Pau and its neighbourhood. It doubtless possesses many advantages both to the healthy and the infirm. Delightful drives and promenades, and the gaiety occasioned by some thousands of persons who have nothing to do, are sufficient attractions for the former; and the abundance, the choice and salubrity of the medicinal springs, are attractive enough to the latter." 225.

House-rent is cheaper than at Pau, and much more so than at any of the other watering-places in the Pyrenees. There are not less than 42 distinct saline sources at Bagnères—all, however, apparently from the same source, and varying but little in their composition. They are by no means powerful springs—not nearly so much so as many that are frequented in England; but they are useful in many functional disorders—and as preparatives for others more potent in the Pyrenees.

The mineral sources at this place are divided into three classes—saline—chalybeate—aud sulphurous. There is but one ferruginous spring, and one sulphureous, properly speaking—both out of the town—the latter, indeed, six miles from Bagnères. The salines are in the town. They are perfectly transparent and limpid—almost inodorous—of mawkish taste, and communicate to the palate a slight chalybeate or rather styptic taste. Their specific gravity is little more than that of distilled water—and indeed they contain little more than a grain of saline matters in the pint. There are 22 springs here, varying in temperature from 81° to 124° of Fahrenheit. Sulphate of lime forms the most preponderating ingredient in these waters—varying from one-tenth to nine-tenths of a grain in the pint. Of the subcarbonate of iron there is just a trace. The "FONTAINE FERRUGINEUSE" is situated ten minutes' walk from Bagnères. The temperature is variable, but never rises above 64°. It contains small quantities of iron, potass, and carbonate of lime.

The SULPHUROUS SOURCE (Labassere) lies six miles from Bagnères, at the feet of the great heights of MONT AIGU. The water is abundant, limpid, without penetrating odour, but the flavour clearly sulphureous. The solid contents are not a quarter of a grain in the pint, and the gaseous very small.

The climate of Bagnères, like that of PAU, is decidedly sedative, and to this place most invalids should resort before they try the other waters of the Pyrenees. The usual physiological effects of these waters is that of a mild stimulant to the mucous surfaces of the stomach and bowels, soliciting a greater secretion of gastric juice, and improving the digestion. If taken in sufficient quantity they act on all the secretions, especially the urine, and relieve congestions. To act on the bowels it will often require two quarts at divided doses in the mornings. As mere alteratives, much smaller quantities will suffice. Dr. Limonnier, who has written well on the Bagnères waters, gives the following resumé of the complaints to which the waters are adapted, internal as well as external.

"In nervous diseases, such as hysteria, hypochondria, palpitations, and nervous spasmodic affections of the stomach, the waters of the Salut in bath are recommended; to which, if there be an atonic habit of body present, the waters of the ferruginous fountain may be added internally; if there be bilious derangement, the waters of Laserre may be alternated with the latter.

"In loss or diminution of voluntary motion, viz., rheumatism, lumbago, sciatica, and paralysis, without injury of the brain, we have the following indications. In the case of an individual of small degree of excitability, the douche and vapour bath, and the baths at a high temperature of Casaux, Dauphin, La Guthière, Petit Bain, and la Fontaine de Laserre; while in one of a nervous temperament, irritable and predisposed to apoplexy and organic congestions, the baths of Foulon, Grand Pré, and No. 3 of Pinac, are indicated. The internal use of the Laserre water is often also associated with this external treatment.

"In pulmonary catarrh, humid asthma, chronic laryngitis, we recommend No. 3 of Pinac, Foulon, Grand Pré, St. Roch, La Guthière, Laserre, the water of Labassere internally, made tepid, mixed with milk, gum-water, or other diluent.

"In excessive discharges from some mucous canals, tepid baths at first, graduated down to those of lower temperature, No. 3 of Pinac, Salut, and lastly des Yeux. Injections also of the waters of Labassere, and of the ferruginous fountain, are practised with great advantage; and these waters are also taken internally in these affections.

"In diseases of the skin with bilious complication, or with any other organic affection, which contra-indicates the use of the sulphurous mineral springs, the waters of Foulon, No. 3 of Pinac, à l'entrée de Laserre, in bath, and the waters of Laserre and Labassere internally, are recommended and taken with advantage.

"In diseases of the abdomen, viz., chronic inflammation of the stomach and bowels, chronic diarrhœa, congestions of the liver and spleen, and chronic inflammation of the liver, we have the following course to pursue. If the affection depend on nervous irritation and a subacute state of inflammatory action, the waters of the Salut may be beneficially used externally and internally; if, on the contrary, it depends on an atonic state, or where there is little excitability in the system, the waters of la Reine, la Fontaine Ferrugineuse, or of Labassere, internally, are the appropriate remedy; if again it be complicated with bilious derangement, without any symptoms of inflammatory re-action, the same waters may be administered in addition to those of Laserre." 248.

### II. WATERS OF CAPBERN.

These are situated only ten miles from Bagnères, and are of a purely saline character. They have, however, acquired a considerable reputation in a certain, but limited class of complaints, to which no small importance is attached. The village of Capbern, 4½ miles from Tournay, contains six hundred inhabitants, and is very pleasantly situated. The waters are considered to be an antidote to sterility, and are therefore visited by all those whose organs of philoprogenitiveness are developed—or the reverse. The waters are limpid, inodorous, of a sweetish taste, and cause a sense of dryness in the throat. Their specific weight scarcely differs from that of distilled water. The temperature is about 76. They flow in great abundance, and disengage a good deal of gas. The solid contents are about eight-tenths of a grain in the pint—and those chiefly common saline matters. The peculiar virtues of Capbern waters (supposing they are possessed of such) must depend on some unknown ingredient—probably a vegetable principle. Their marked stimulus on the uterine system, has led the physician to suppose that something analogous to the ergot of rye may be infused in these springs. Although they are said to be very useful in many disorders—especially in congestions of various organs—yet their physiological mode of action is that of exciting the uterine vessels in women and the hæmorrhoidal in men. They are taken internally and used as baths.

But as we do not wish to ruin Ems, Liebenstein, and Schwalbach, we shall take leave of Capbern, not doubting that some of our readers will try the efficacy of the *philoprogenitive spring* of the Pyrenees.

### III. BARREGES.

This is the Lion of the Pyrenees—in the same way as the "OLD WELL" is the Lion of Harrogate. It is distant from PAU about 47 miles, in a South-eastern direction—the road being as good as the average of turnpikes in England. The diligence runs every second day, between an early breakfast and a late dinner. Commodious voitures may be hired at 20 francs a day. At Lourdes we strike at once into the Pyrenees, and the scenery here is of the first order—romantic and sublime.

Barréges itself is a village capable of containing, in the season, some twelve hundred souls. It is 4000 feet above the level of the sea—composed of one street—built on the shelf of a mountain, overlooking a noisy torrent—and surrounded by bleak mountains, usually canopied by mist. Voila BARREGES. It is evident that the inducements must be cogent ones, which could neutralize the disadvantages of so triste and ungenial an abode! But what will not man undergo in pursuit of health as well as of wealth? The waters of Barrèges are comprised in three principal sources—the *hot source*—the *temperate*—and the *tepid*. These supply 17 baths, two douches—and two large basins—one for the military, and one for the poor. There is also a source reserved for the drinkers. The waters of all these sources are clear, limpid, and exhale an odour of rotten eggs. Their taste is mawkish, nauseous, and oleaginous. Their surface is covered with a thin pellicle, of unctuous appearance, and they deposit this glairy substance on the sides of the baths, &c. It is called *Barregine*. The supply is prodigious. Their temperature ranges from 110° to 84° of Fahr. The solid ingredients are so small, that a pint of the water does not contain a tenth part of a grain!! It is the sulphuret of soda that gives them their sulphureous smell—being about one twenty-fifth part of a grain in the quart. The following is the carte des maladies for which the waters of Barrèges are chiefly employed, viz.—1. Diseases of the skin, with their varieties,—squamous, pustular, papular; 2. Affections of muscular, fibrous, tendinous, and membraneous tissues, comprising rheumatalgia, lumbago, sciatica, articular rheumatism, muscular retractions, anchylosis, white swelling, articular enlargements; 3. Deep-seated irritation, arising from the presence of foreign bodies, collections of matter, carious bones; 4. Scrofulous and ill-conditioned sores, and fistulous ulcers.

Four or five miles from Barrèges is situated ST. SAUVEUR, whose waters are feebler than those of the former, and are used as preparatory sometimes to the stronger springs.

### IV. CAUTERETS.

In another magnificent gorge of the Pyrenees rise some celebrated sources of mineral waters—the CAUTERETS. The town is situated in a solitary but picturesque spot, and contains a stationary population of some 800 souls—but capable of accommodating a thousand visitors. There is a great variety of mineral springs here, and the sheltered site permits of Winter as well as Summer residence.

Cauterets possesses eleven independent sources, pent up in a small compass, yet exhibiting specimens of almost all the sulphurous waters scattered over the Pyrenees. There are sources even more powerful than the waters of Barrèges—waters possessing the virtues of the Eaux-Chaudes—Eaux-Bonnes—springs shading from the most powerfully stimulant, down to the mild and soothing sulphurous waters of St. Sauveur. Cauterets is more than a thousand feet lower than Barrèges—is better sheltered—has a less keen air—and not near so much subject to fogs. The spring that is most used here is the RAILLERE, of 104 degrees of temperature, and not containing more than a tenth of a grain—if so much—of solid matters in a pint of water. There is about a hundredth part of a grain of sulphuret of sodium in the pint. Some of the sources are as high as 131° of Fahrenheit. This source is celebrated for disorders of the mucous membranes—especially those of the chest. The number of candidates of this class, and the number of cures performed, seem to attest the powers of this water. It is situated a mile from the town, and the ascent to it is fatiguing to a debilitated person. But the hardy mountaineers trot him up briskly in a sedan-chair. The drinking and bathing processes are carried on between 4 and 10 o'clock in the morning—sometimes through the whole night.

There is one passage in Dr. Taylor's work which shews a considerable drawback on the virtues of these waters in certain obscure pathological conditions of the lungs—namely, where there are *miliary tubercles*—a condition which we fearlessly maintain to be incapable of accurate diagnosis by the stethoscope—whatever auscultators may say to the contrary. But our author avers that, whenever these tubercles take on "*a state even of incipient activity,*" the waters of La Raillère are dangerous agents. How difficult to say the precise time when tubercles *begin,* as it were, to shoot out from their nascent and latent condition! It appears evident, however, that the waters in question are very serviceable in affections of the mucous membranes—laryngeal, tracheal, and bronchial—and for that we must be thankful.

### V. EAUX-BONNES AND EAUX-CHAUDES.

These are waters of the mildest sulphureous kind—and might be used in almost all diseases—or all stages of any disease. They are situated at the foot of the great Pyrenean chains, about 26 miles from Pau, and within a short distance of one another. The road, which is excellent, runs directly South from Pau. The famous little watering-place of Eaux-Bonnes is seen crouching in a cul-de-sac, under the protection of mountains 8000 feet high. There are not more than twenty houses in the village; yet they accommodate five or six hundred invalids annually!

The village is rather more than 2000 feet above the level of the sea. The air is pure and fresh—and less agitated by winds than in most other parts of the Pyrenees—and consequently better suited to pulmonary invalids than any of the other sulphurous springs in these mountains. There are five distinct sources in Eaux-Bonnes. That for drinking is the Source Vieille, at a temperature of 88.° The Source de la Douche has a temperature of 91°. A pint of the waters contain about three-fifths of a grain of solid contents, with sulphuret of sodium, to which the odour is due. These waters have gained considerable reputation in the cure of pulmonary affections. In 1840, 2,800 persons took the waters and baths here, and 50,000 bottles of the water were taken away.

### VI. EAUX-CHAUDES.

This petty village is situated in a gorge of the Pyrenees, with some dozen or two of houses and bad accommodations. The waters are called hot, on the principle of *lucus a non lucendo,* for the highest temperature is 97½, or not quite blood-heat. Their reputation depends chiefly on the wonderful cures which they are said to perform in obstinate chronic rheumatism. They are sulphureous like the Eaux-Bonnes, and contain about a pinch of saline matters in the gallon.

We may conclude by expressing our opinion that Dr. Taylor has given a fair, and we think impartial exposition of the Climate of Pau and the Waters of the Pyrenees.

